# Older adults’ perceptions and informational needs regarding frailty

**DOI:** 10.1186/s12877-018-0741-3

**Published:** 2018-02-13

**Authors:** Nancy L. Schoenborn, Sarah E. Van Pilsum Rasmussen, Qian-Li Xue, Jeremy D. Walston, Mara A. McAdams-Demarco, Dorry L. Segev, Cynthia M. Boyd

**Affiliations:** 1 The Johns Hopkins University School of Medicine, 5200 Eastern Avenue, Mason F. Lord Building Center Tower, Room 711, Baltimore, MD 21224 USA; 2 The Johns Hopkins University School of Public Health, Baltimore, MD 21287 USA

**Keywords:** Frailty, Older adults, Communication, Qualitative research

## Abstract

**Background:**

Frailty has been recognized as an important medical syndrome in older adults. Growing literature supports the clinical application of frailty but US older adults’ perceptions of frailty have not been explored. We aim to examine perceptions and informational needs about frailty among older adults.

**Methods:**

This was a qualitative study involving focus groups of community-dwelling older adults with diverse age and frailty status. We explored participants’ beliefs and knowledge about frailty and informational needs about frailty as a medical syndrome.

**Results:**

The participants’ mean age was 76.3. Of the 29 participants, 21 (72%) were female, and 21 (72%) were white. We identified three major themes: 1) Older adults’ perceptions of frailty differed from the definition used in medical literature; they often perceived a psychological component to being frailty and some were skeptical of the syndromic definition based on multiple symptoms. 2) Compared to participants who were non-frail or pre-frail, participants who were frail were more receptive to discussing their frailty status with clinicians; 3) Participants wanted know about how to treat or prevent frailty and the risks associated with being frail. Many participants felt that these information can be conveyed without necessarily using the specific term “frail”, which they perceived to have a negative connotation.

**Conclusions:**

Older adults, especially those who are frail, may be interested to discuss frailty as a medical syndrome. However, negative perceptions are associated with the term “frail” and may be a barrier to clinical application of frailty. Further research is needed to understand acceptable ways for communicating about frailty in clinical practice.

**Electronic supplementary material:**

The online version of this article (10.1186/s12877-018-0741-3) contains supplementary material, which is available to authorized users.

## Background

Physical frailty, defined as a medical syndrome consisting of specific physical symptoms [1, 2], has been recognized as an important entity in older adults that is predictive of multiple adverse outcomes including falls, hospitalization, functional dependence, and death [2–4]. There is growing literature supporting the clinical application of frailty, including recommendation to regularly screen older adults for frailty [4]. Frailty assessment is also increasingly used to stratify risk and inform clinical decisions in various surgical procedures, cancer treatment, and kidney transplantation [5–7].

How the frailty syndrome and its prognostic implications should be communicated with patients in the clinical setting has not been explored. Studies in the United Kingdom (UK) and Netherlands show that older adults view the term “frailty” as a negative label and resist being labeled as frail. [8–11] Studies also suggest that older adults often understand the term “frailty” using lay definitions, rather than as a medical diagnosis, where the term is often associated with age-related stereotypes and negative psychological and social states such as dependency and fear [8–13]. There is no empiric data on how older adults in the United States (US) perceive frailty, what information they would want to know about frailty, or how they prefer to receive such information. This is a critical knowledge gap that needs to be addressed in order to more widely apply frailty in clinical practice to improve the care of older adults.

Because relatively little is known about this area, we use qualitative methods in this study to explore the range of perspectives from older adults to generate hypotheses. We aim to examine existing beliefs and knowledge about frailty, views about frailty as a medical syndrome, and informational needs and communication preferences for discussing frailty among community-dwelling older adults across the spectrum of frailty.

## Methods

### Design and study setting

This was a qualitative study in which focus groups lasting 55–85 min were conducted with community-dwelling older adults (65 years or older). Participants were recruited from a registry of older adults in the local communities who have expressed interest in research studies and have previously given consent to be contacted for studies related to aging and frailty if they are qualified. This project was approved by a Johns Hopkins School of Medicine institutional review board.

### Subjects and recruitment

The registry of older adults was started in 2007 as part of the clinical translational unit of the Johns Hopkins Older Americans Independence Center. The registry is composed of volunteer community-dwelling older adults who are 65 years or older, recruited from the Beacham Ambulatory Care Center (Geriatric Medicine Clinic), the Bayview General Internal Medicine Outpatient clinic, community health fairs and outreach events focused on older adults throughout the Baltimore metropolitan area. In addition, some individuals are recruited through Baltimore or local neighborhood newspaper advertisements. After informed consent, all registry members receive a baseline comprehensive assessment of their health status, including a cognitive assessment using the mini-mental state examination (MMSE) and a physical frailty assessment using the Fried criteria [1, 2]. The frailty assessment includes measures of hand grip strength, gait speed, self-reported weight loss, low physical activity, and exhaustion. In each of the five measurement domains, a person is assigned a score of 1 if predefined cut-off points are met, or a score of 0 if the cut off points are not met, with a total score range of 0–5. Those with a score of 3 and above are considered frail, a score of 1–2 as pre-frail, and a score of 0 as non-frail. The cognitive assessment and the frailty assessment may be subsequently repeated as part of specific research studies in which registry members participate.

All registry members were eligible to participate in this study if they were English-speaking and able to provide informed consent. Recruitment occurred by phone and was stratified by the most recently available frailty status. Each focus group was restricted to participants of the same frailty status because within-group homogeneity was thought to facilitate more free discussion. We aimed to recruit at least one focus group with participants who were non-frail, pre-frail, and frail, respectively, and to oversample the frail participants because they are the most relevant for our study question. Recruitment stopped when theme saturation in the data was reached [14], as described in section below (Data collection and analysis). Each participant was provided a $50 gift card, and parking voucher as needed.

### Focus group discussion guide

The discussion guide (Additional file 1) was developed and iteratively revised during pre-testing with 8 older adults who were not included in the study. At the beginning of the focus group, we explored the participants’ knowledge and perceptions of frailty. We then provided a brief overview of the Fried criteria [1, 2]; we mentioned that frailty is considered a medical condition, similar to hypertension for example, and that not everyone who gets old will necessarily become frail. We elicited reactions and questions from the participants; we specifically asked if the participants would like their clinician to discuss their frailty status with them, whether the clinician should discuss the long-term risks associated with frailty, and what additional information they would like to know about frailty.

### Questionnaire

A structured questionnaire prior to the focus group collected information on self-reported health status [15], health literacy [16], numeracy [17], and trust in the clinician (“All in all, you have complete trust in your doctor.” 1 = strongly disagree, 5 = strongly agree) [18]. Other demographic characteristics had already been collected as part of the registry.

### Data collection and analysis

Two investigators (NS and SR) with prior qualitative research experiences conducted the focus groups in person between May and August 2016. The investigators had no direct clinical relationship with any participant. Focus groups occurred in a private conference room at the Johns Hopkins Bayview Medical Center. All focus groups were audio-recorded and study team members also took notes during the discussion. The audio-recordings were then transcribed verbatim and analyzed using Atlas.ti textual data analysis software [19]. The notes and the transcripts were continuously reviewed and assessed for the emergence of new ideas or themes; data collection continued until no new ideas were emerging and theme saturation was reached. [14] Standard techniques of directed qualitative content analysis were used to code the transcripts. [14, 20] A preliminary coding scheme based on the focus group discussion guide was iteratively refined and applied to analyze the data using the constant comparative approach [14, 21]. Open coding procedures allowed new theme identification in addition to the established scheme. Revisions to the coding scheme were applied to all previously coded transcripts. Two investigators (NS, SR) independently coded all transcripts. Differences were reconciled by consensus until 100% agreement was reached. Content analysis generated major themes and sub-themes.

## Results

Twenty-nine older adults participated in four focus groups (Table 1). The participants’ average age was 76.3 with standard deviation (SD) of 7.8 years. Of the 29 participants, 21 (72%) were female; 21 (72%) were white. Two focus groups consisted of 12 participants who were frail; one focus group consisted of 8 participants who were pre-frail; one focus group consisted of 9 participants who were non-frail. The average time since last frailty assessment was 1.5 years, ranging from under 3 months to over 6 years. The time since frailty assessment was shorter for non-frail and pre-frail participants (average 0.4 and 1.0 years respectively) than for frail participants (average 2.8 years). The most recent MMSE scores for the participants ranged from 25 to 30 with average of 28.2.

**Table 1:** Participant Characteristics (*N* = 29)

Characteristic	Data
Age, mean (SD), year	76.3 (7.8)
Female sex, No. (%)	21 (72.4%)
Race, No. (%)	
- White	21 (72.4%)
- African American	7 (24.1%)
- Other	1 (3.4%)
Frailty status at time of recruitment, No. (%)	
- Non-frail	9 (31.0%)
- Pre-frail	8 (27.6%)
- Frail	12 (41.4%)
Time since frailty assessment, mean (SD), year	1.5 (1.7)
Mini-mental state examination (MMSE) (possible range 0–30)	28.2 (1.4)
Time since MMSE, mean (SD), year	0.6 (0.8)
Self-reported health, No. (%)	
- Excellent or very good	17 (58.6%)
- Good	9 (31.0%)
- Fair or poor	3 (10.3%)
Educational level, No. (%)	
- Completed high school	6 (20.7%)
- < 4 year college	7 (24.1%)
- College graduate or post-graduate degrees	16 (55.2%)
Health literacy [16] (possible range 3–15)	13.9 (1.6)
Numeracy [17] (possible range 3–18)	14.0 (3.5)
Trust in clinician [18] (possible range 1–5)	4.2 (0.8)

Qualitative content analysis revealed three major themes with sub-themes; these are presented below and illustrated using representative quotes.

### Theme 1. Older adults’ perception of frailty differed from the definition used in medical literature

Prior to providing a definition of frailty, the older adults’ baseline perception of what it means to be “frail” involved multiple physical symptoms, many of which are consistent with the Fried criteria (Table 2) [1, 2]. However, their perception also differed in several important aspects and these differences in perspective persisted even after providing a definition of the Fried criteria for frailty.

**Table 2:** Older adults’ perceptions of frailty

Perception domains	Example
Frailty as related to age	“*I’ve got family members who have been [frail] basically because of age.”*
Frailty as related to physical symptoms	
Lack of strength or energy	*“Someone [who] doesn’t have strength or energy.”*
Decreased activity	*“Their ability to do the things that they once did are gone.”*
Trouble walking	*“I have experience with elderly people being frail and not being able to walk too far or having to use a walker.”*
Weight loss	*“She’s very frail, she weight maybe 110 and she was weighing 140…. So she’s pretty frail right now.”*
Low weight	*“Lack of physical weight… if a wind would come they might get blown away… I associate [frailty with] very thin people.”*
Tendency for falling	*“When … they have difficulty and they are fall risks … they become frail.”*
Weak bones	*“I think of people with brittle bones who if they fell could break the bones.”*
Physical symptoms that are perceived to *not* be related to frailty	
Lack of energy	*“I think as you get older there’s certain things you can’t do, like lack of energy, you know you used to be able to do certain things and then all of a sudden you get tired and you feel like taking a nap. I’m 80 years old and I feel like if I want to take a nap I’m entitled. That isn’t frail. It’s getting older.”*
Trouble walking	*“I have a condition with my spine where I can’t walk very well or for any distance but to me in my mind that doesn’t make me frail necessarily, it’s just a limitation.”*
Frailty as related to subjective feeling or psychological state	
A subjective feeling	“*I felt frail when I had my first child.”*
A mental state	*“It’s a state of mind…you know for the individual to decide if he or she feels that frailty.”*
Mental state causes frailty	*“If you consider yourself frail, you are gonna be frail.”*

First, older adults associated frailty with increased age, even though we explained that not everyone who gets old will necessarily become frail. For example, one participant said:

*“A frail person was an older person, and you know, they really couldn’t maneuver and you would have to help the person because they were frail and old.”*

Second, the participants often mentioned a psychological component to being frail in addition to having physical symptoms.

*“I think frailty is a state of mind… thinking of yourself as frail then you gonna have some subsequent frail behavior.”*

After providing a definition of frailty to be a medical syndrome consisting of symptoms in five domains: weakness, decreased physical activities, decreased energy, slow walking, and weight loss, a number of participants expressed resistance to the presented definition. Some disliked having a definition based on multiple symptoms domains; one person said:

*“Frailty is a generalization and I don’t think it has really any place in the medical conversation….whatever the element that goes into making up frailty ought to be discussed, but the generalization of frailty I don’t think is helpful at all.”*

Others objected to specific symptom domains that are included in the definition:

*“Just because I can’t walk as fast as I did two years ago does that mean that I’m frail? That seems unfair really.”*

### Theme 2: The frail participants were more receptive to discussing frailty than non-frail or pre-frail participants.

#### Non-frail and pre-frail participants

When we asked how the participants would react if their clinician offered to discuss whether or not they are frail, several of the non-frail and the pre-frail group participants were vehemently against the suggestion, saying:

*“Flatly no.”*

*“[I would] get another doctor. I’m dead serious.”*

*“That’s a very bad approach it seems to me”.*

Some felt that the patients would already be self-aware of whether or not they are frail and would not need to be told:

*“A doctor doesn’t have to even label it, a person would know if they are frail.”*

These participants who were not frail also perceived frailty as a terminal outcome where discussion would be too late:

*“I see being frail as the end of the line, before you get frail you should be eating properly, exercising…I don’t know how once you get frail you are gonna bring yourself back up.”*

Many participants viewed the term “frail” as a very negative term:

“*the whole attitude [is one] … of defeat as soon as you see that word.”*

Some participants believed that telling someone that he or she is frail would then lead to deterioration of their status:

*“When a physician would say to somebody [that he or she is frail]…would that have any detrimental effect on the individual of start becoming more frail and start acting more frail?… Because psychologically that seeds been planted…, [the individual may think]: ‘I’m frail so I guess I’m just gonna have to sit in this chair and watch television 24 hours a day.’”*

#### Frail participants

On the contrary, both of the focus groups consisting of frail participants were much more amenable to discussing frailty status with clinicians than the non-frail and the pre-frail participants.

*“We want to know what’s going on and how to treat it and we are not afraid to hear these words, we get more upset that you didn’t tell us.”*

However, even among these, only a subset of them welcomed the specific term of “frailty”.

*“I don’t find anything wrong with the word frailty.”*

Others felt that the important information can be conveyed without using the specific term of “frailty”, and preferred not using the specific term of “frailty”.

*“Don’t use the word, in other words instead of saying: 'mister, you are frail', I’d say: 'mister, hold onto the banister when you are going downstairs and watch where you are walking', in other words give them specifics but don’t label them.”*

We elicited suggestions for how clinicians can discuss frailty with patients from all participants; these are summarized in Table 3; these included emphasizing hope, making clear distinction that “frailty” is a medical diagnosis as opposed to a general description, involving family members in the discussion, providing written information, and individualizing the discussion. For example, one participant said:

*“[Discuss] whatever is appropriate for [this patient], if [this patient] doesn’t ride a bicycle, you don’t have to tell him to stay off the bicycle.”*

**Table 3:** Older adults’ suggestions for how clinicians can discuss frailty with patients

Suggestions	Example
Provide hope	*“They are frail but give them the message that there’s definitely hope and here’s some things that they can do. Hope is the most important thing.”*
Emphasize “frailty” as a medical diagnosis	*“I’d find a better way to …make it sound more medical… indicate that it definitely is a diagnosed condition.”*
Avoid the term “frailty”	*“It’s not like it’s a new word, it’s been in the vernacular for a long time and I think different people have different thoughts about it. I don’t think I’d say to anybody: ‘you are frail.’”*
Involve others in the conversation	*“Some news you can’t really handle by yourself…so you need a supportive network to get you through the situation.”*
Written information	*“Some written material with suggestions that’s really thought through is also helpful for the patient to take home.”*
Tailor the discussion to the individual	*“Everybody’s frailty is different. It sounds like there’s so many things that you can be labeled as frail…I think they have to deal with it like an individualized frailty.”*

### Theme 3. Informational needs about frailty

Among all groups, when asked what information they would like to know about frailty from their clinicians, participants described that the most important information they wanted to know was what can be done to prevent or improve their condition.

*“[I want to know] what do you recommend… to mitigate some of the frailty and maybe get less frail or more healthy.”*

Those who may already be frail wanted to know how to adapt to being frail:

*“I look forward to hopefully getting to be really old and becoming less resilient and less able to do things, what do I replace it with? What will I be able to do in my life that maybe I’ll do more of.”*

The participants were open to hearing the risks of functional decline and complications that are associated with being frail. Even the non-frail and pre-frail patients thought hearing about the potential downstream risks would motivate someone to take actions to prevent becoming frail. One participant said:

*“I think they should discuss risks because everyone should be prepared with what is going on… with risks come preparation.”*

## Discussion

Building on the small body of literature that explored older adults’ attitudes about frailty in other countries [8–13], this is the first project that characterizes US older adults’ perceptions of frailty. Consistent with findings in the UK and the Netherlands [8–10], we found that our participants often associated negative connotations with the term “frail” and most did not like being labeled as such. We compared older adults’ perceptions of frailty to the Fried definition for frailty syndrome that is commonly used in the medical literature [1, 2], which has not been previously examined. We found that although many older adults attributed physical symptoms to being frail, there were two important differences between the participants’ concept of frailty and the Fried definition. First, the participants associated frailty as an inevitable part of aging. Second, the participants described a mental or psychological component to frailty, both as a cause of frailty (believing one to be frail would then lead one to become frail) and as a symptom (a frail person would feel frail). These difference in perceptions persisted even after providing the Fried definition for frailty.

We examined the perceptions of frailty among groups with different frailty status, which has not been explored in previous studies. We were surprised to find that the more frail participants were more receptive to discussing frailty, as compared to the non-frail and pre-frail participants. This may be because that actually living with the symptoms of frailty helped these participants accept the diagnosis without much emotionality whereas for the healthier participants it may be more anxiety-provoking to consider being frail, especially when it was viewed as a permanent, irreversible condition. However, even among the frail participants who were amenable to discussing frailty, many of them still preferred not to use the specific term of frailty to describe their condition. These findings demonstrate the challenge of using terms such as frail and frailty, which are common in the public lexicon, to describe specific, complex scientific constructs that describe related but not entirely identical concepts than the lay vocabulary. An important next step is to examine the potential heterogeneity in views by frailty status in a larger population. Our results also suggest that negative perceptions around the term “frail” may be a barrier to the clinical application of frailty as a syndrome; a different term to represent the concept of physical vulnerability may be preferable to patients and should be examined in future studies.

This is the first project to describe older adults’ informational needs about frailty. The most important informational need among our participants relates to intervention, i.e. what can be done to prevent or improve frailty status. There is a small but growing body of evidence that interventions such as comprehensive geriatric assessment, exercise, nutrition, and prehabilitation may be effective in reducing the level of frailty in pre-frail or frail individuals; less research is available on interventions to prevent frailty [4, 22]. In light of the limited information that is available regarding an aspect of frailty that the patients would most want to know, it is questionable whether it may be premature to implement routine screening for physical frailty in clinical practice at the present time [23]. Furthermore, given the negative reactions to the term “frailty” and that the interventions are not exclusive to frailty, it is conceivable that clinicians may make the appropriate recommendations without having to explicitly discuss frailty. More research is needed to identify targeted interventions that can prevent or reverse frailty. Interventions are also needed to help older adults who are frail to accept and adapt to frailty.

This study has several limitations. First, it was conducted with participants from a local registry and may not represent experiences of older adults elsewhere. In addition, the study did not include participants with significant cognitive deficits. This study was not designed to be representative of all older adults, but rather to gain in-depth perspectives about a topic where little was previously known. Second, the study design relied on self-report and the results are prone to social desirability biases. Third, we focused on the participants’ perspectives and reactions around the Fried definition of frailty and did not test other definitions that exist in the literature [24]. However, our participants’ perceptions of frailty were consistent with findings of other studies that used different definitions of frailty [8–10]. Lastly, for most of the frail participants, several years have lapsed since their frailty assessment and their frailty status may have changed. We nonetheless posit that these participants who have ever been frail offer valuable and different perspectives than those who have never been frail.

## Conclusions

In summary, we found that older adults have negative perceptions of the term “frailty” and have different understandings of the term than the definition that is used in the medical literature. Those who are frail are amenable to discussing frailty as a medical syndrome with their clinicians but preferred doing so without the label of frailty. They also suggested that the most important informational need is around interventions. These results highlight that despite the growing the body of research around frailty, there remain important gaps to operationalizing physical frailty as a medical diagnosis. Specifically, better understanding acceptable ways to communicate the concept of frailty syndrome to patients and developing effective interventions to treat frailty are two critical areas that need to be addressed before the frailty syndrome is widely implemented as a clinical entity into routine practice.

## Additional file

Additional file 1: Focus group discussion guide: These were the questions used to guide discussions during focus groups. (DOC 36 kb)

## Abbreviations

MMSE: Mini-mental state examination
SD: Standard deviation
UK: United Kingdom
US: United States
