# Supplementary material for: Older adults’ perceptions and informational needs regarding frailty
Source: BMC Geriatr. 2018 Feb 13;18:46. doi: 10.1186/s12877-018-0741-3 (PMC5809948; doi:10.1186/s12877-018-0741-3)
Supplement: Supplementary file 1 — Focus group discussion guide: These were the questions used to guide discussions during focus groups. (DOC 36 kb) [file 12877_2018_741_MOESM1_ESM.doc]

**Focus Group Discussion Guide**

Thank you all for being here for this study. We will be spending the session talking about something called frailty and how doctors can better talk to older adults about this. We are interested in your opinions, there is no right or wrong answer.

1. When you hear the word “frail” or “frailty”, what does that mean to you? What comes to mind? Have you heard the word before? If you have heard the word before, where/in what context did you hear about it?
2. Medical professionals define frailty as a chronic health condition, like high blood pressure or diabetes, that can slowly develop over time. People who are frail have a harder time bouncing back or recovering after they get sick or hurt, like from an infection or a surgery. Frailty is more common as people get older but is not part of normal aging; it is not something you always get as you get older. Common signs and symptoms of frailty include weight loss, walking slower, weaker strength, feeling more exhausted, less physical activities.

What questions do you have about what I just said? Can you think of someone that you know who may be frail?

1. Would you want to be told by a doctor whether or not you are frail? If you were told by your doctor that you are “frail” or have “frailty”, how would that make you feel? Some people do not like to be called “frail”, do you have any suggestions for other ways to discuss this information?
2. What would you want to know from the doctor about being “frail”?
3. We know that if a Mr. Jones is “frail”, he may be more likely to get weaker over time, or may not live as long, and that he is at higher risk for side effects from certain medications or procedures. But there are things we can do to help Mr. Jones, such as exercise, healthy diet, making sure he’s on the right medications, and avoiding procedures that may be too risky.

This type of information may be hard for Mr. Jones or his family members to hear. Do you think that patients would want to know these risks that come with being frail, like getting weaker more quickly over time, may not live as long, higher risk for side effects? Or do you think doctors should make the appropriate recommendations without discussing these risks?

Do you think the doctor should discuss all of these risks or only some? (for example, talking about Mr. Jones getting weaker because he’s frail may be more difficult to hear than about Mr. Jones having higher risk of side effects from medications) How would knowing the risks be helpful? Would knowing about the risks make someone more motivated to make a positive change about their health?

1. Pretending that you were the doctor, how would you tell Mr. Jones about being “frail” and the risks that come with being frail? How could these information be said without being too negative?
2. Is there anything we should have talked about but didn’t?
